# Supplementary material for: Structurally reorganized Wmk lineage with distinct genomic context and restricted distribution in Wolbachia
Source: G3 (Bethesda). 2026 Aug 27;16(11):jkag234. doi: 10.1093/g3journal/jkag234 (PMC13732484; doi:10.1093/g3journal/jkag234)
Supplement: Supplementary file 1 [file jkag234_supplementary_data.zip › Supplemental_Data1_G3-2026-407105.pdf]

List of *Wolbachia* genomes used in the study.

accesion_number	host_order	host_name	supergroup	assembly_type	contig_number	genome_size_bp
GCA_947251615	Coleoptera	*Apoderus coryli*	A	circular	1	1535196
GCA_947250775	Coleoptera	*Rhinocyllus conicus*	A	circular	1	1703908
GCA_947251755	Coleoptera	*Philonthus cognatus*	A	circular	1	1811324
GCA_947179345	Coleoptera	*Philonthus cognatus*	B	linear	2	1680410
GCA_947250015	Diptera	*Acrocera orbiculus*	A	circular	1	1383045
GCA_947251895	Diptera	*Bombylius major*	A	circular	1	1556587
GCA_947251425	Diptera	*Protocalliphora azurea*	A	circular	1	1434286
GCA_947251865	Diptera	*Protocalliphora azurea*	B	circular	1	1550351
GCA_947251515	Diptera	*Sicus ferrugineus*	A	circular	1	1273941
GCA_947251955	Diptera	*Coremacera marginata*	A	circular	1	1387347
GCA_947250625	Diptera	*Cheilosia soror*	A	circular	1	1368131
GCA_947251925	Diptera	*Epistrophe grossulariae*	A	circular	1	1483878
GCA_947251435	Diptera	*Epistrophe grossulariae*	A	circular	1	1367569
GCA_947250575	Diptera	*Episyrphus balteatus*	B	circular	1	1402289
GCA_947250695	Diptera	*Eupeodes latifasciatus*	B	circular	1	1274269
GCA_947251465	Diptera	*Melanostoma mellinum*	B	circular	1	1875946
GCA_947250735	Diptera	*Sphaerophoria taeniata*	A	circular	1	1354583
GCA_947250665	Diptera	*Volucella inflata*	A	circular	1	1502845
GCA_947250765	Diptera	*Gymnosoma rotundatum*	A	circular	1	1431527
GCA_947251545	Diptera	*Anomoia purmunda*	A	circular	1	1485792
GCA_947251675	Diptera	*Merzomyia westermanni*	A	circular	1	1295458
GCA_947251645	Diptera	*Bibio marci*	A	circular	1	1355352
GCA_947179435	Diptera	*Cheilosia soror*	A	linear	2	1493041
GCA_947179415	Diptera	*Platycheirus albimanus*	A	linear	6	1174338
GCA_947179355	Diptera	*Sphaerophoria taeniata*	B	linear	3	1627501
GCA_947179175	Diptera	*Volucella inflata*	A	linear	2	1346192
GCA_947251635	Hemiptera	*Icerya purchasi*	A	circular	1	1372034
GCA_947251765	Hymenoptera	*Andrena dorsata*	A	circular	1	1366845
GCA_947251685	Hymenoptera	*Andrena dorsata*	A	circular	1	1533669
GCA_947250565	Hymenoptera	*Andrena haemorrhoa*	A	circular	1	1494006
GCA_947250795	Hymenoptera	*Nomada fabriciana*	A	circular	1	1438975
GCA_947251915	Hymenoptera	*Hylaeus communis*	A	circular	1	1526906
GCA_947251695	Hymenoptera	*Ectemnius continuus*	A	circular	1	1299221
GCA_947251665	Hymenoptera	*Trypoxylon clavicerum*	A	circular	1	1287268
GCA_947250585	Hymenoptera	*Lasioglossum morio*	A	circular	1	1282688
GCA_947251705	Hymenoptera	*Lasioglossum morio*	A	circular	1	1270694
GCA_947250685	Hymenoptera	*Sphecodes monilicornis*	A	circular	1	1359653
GCA_947251965	Hymenoptera	*Sphecodes monilicornis*	A	circular	1	1141923
GCA_947251485	Hymenoptera	*Scambus nigricans*	A	circular	1	1150903
GCA_947251555	Hymenoptera	*Macropis europaea*	A	circular	1	1439111
GCA_947251795	Hymenoptera	*Anoplius nigerrimus*	A	circular	1	1345812
GCA_947251845	Hymenoptera	*Athalia cordata*	B	circular	1	1345842
GCA_947251725	Hymenoptera	*Tiphia femorata*	A	circular	1	1330166
GCA_947250785	Hymenoptera	*Ancistrocerus nigricornis*	A	circular	1	1265149
GCA_947179565	Hymenoptera	*Andrena hattorfiana*	A	linear	3	1409226
GCA_947251945	Hymenoptera	*Lasioglossum lativentre*	A	linear	1	1306351
GCA_947179495	Hymenoptera	*Lasioglossum malachurum*	A	linear	2	1380835
GCA_947179405	Hymenoptera	*Lasioglossum malachurum*	A	linear	2	1363507
GCA_947250495	Hymenoptera	*Lasioglossum morio*	A	linear	1	1284395
GCA_947179545	Hymenoptera	*Sphecodes ephippius*	A	linear	2	1317045
GCA_947179505	Hymenoptera	*Dolichovespula media*	B	linear	3	1685384
GCA_947250755	Lepidoptera	*Agriphila straminella*	B	circular	1	1856314
GCA_947251835	Lepidoptera	*Apotomis turbidana*	B	circular	1	1525756
GCA_947250705	Lepidoptera	*Archips podanus*	B	circular	1	1806407
GCA_947251935	Lepidoptera	*Calamotropha paludella*	A	circular	1	1594318
GCA_947251525	Lepidoptera	*Calamotropha paludella*	A	circular	1	1378301
GCA_947250595	Lepidoptera	*Campaea margaritaria*	B	circular	1	1507682
GCA_947251825	Lepidoptera	*Camptogramma bilineatum*	B	circular	1	1668206
GCA_947250515	Lepidoptera	*Catoptria pinella*	B	circular	1	1667357
GCA_947251805	Lepidoptera	*Celastrina argiolus*	B	circular	1	1329046
GCA_947250525	Lepidoptera	*Colias croceus*	B	circular	1	1396841
GCA_947251605	Lepidoptera	*Emmelina monodactyla*	B	circular	1	1385743
GCA_947250805	Lepidoptera	*Endotricha flammealis*	A	circular	1	1691857
GCA_947251735	Lepidoptera	*Endotricha flammealis*	B	circular	1	1747616
GCA_947251745	Lepidoptera	*Epagoge grotiana*	A	circular	1	1431999
GCA_947251775	Lepidoptera	*Epagoge grotiana*	A	circular	1	1781589
GCA_947251475	Lepidoptera	*Epirrhoe alternata*	A	circular	1	1197406
GCA_947250645	Lepidoptera	*Eucosma cana*	B	circular	1	1407592
GCA_947250535	Lepidoptera	*Euphydryas aurinia*	B	circular	1	1802478
GCA_947251595	Lepidoptera	*Eupithecia tripunctaria*	A	circular	1	1478720
GCA_947251975	Lepidoptera	*Hylaea fasciaria*	B	circular	1	1307822
GCA_947251875	Lepidoptera	*Lycaena phlaeas*	B	circular	1	1312316
GCA_947250815	Lepidoptera	*Nymphalis c-album*	B	circular	1	1381694
GCA_947250465	Lepidoptera	*Nymphalis c-album*	B	circular	1	1236246
GCA_947251495	Lepidoptera	*Pammene fasciana*	B	circular	1	1266982
GCA_947250555	Lepidoptera	*Pandemis corylana*	B	circular	1	1510264
GCA_947250635	Lepidoptera	*Parapoynx stratiotata*	B	circular	1	1545403
GCA_947250725	Lepidoptera	*Pararge aegeria*	B	circular	1	1332905
GCA_947250505	Lepidoptera	*Phalera bucephala*	A	circular	1	1271809
GCA_947251565	Lepidoptera	*Pheosia gnoma*	A	circular	1	1353731
GCA_947251655	Lepidoptera	*Pheosia tremula*	B	circular	1	1268782

GCA_947250605	Lepidoptera	*Pyrgus malvae*	B	circular	1	1544873
GCA_947250615	Lepidoptera	*Rhopobota naevana*	B	circular	1	1771993
GCA_947250655	Lepidoptera	*Watsonalla binaria*	B	circular	1	1651336
GCA_947250675	Lepidoptera	*Xestia c-nigrum*	B	circular	1	1372378
GCA_947179465	Lepidoptera	*Agriphila tristella*	B	linear	2	1312870
GCA_947250485	Lepidoptera	*Aporia crataegi*	B	linear	1	1310109
GCA_947250475	Lepidoptera	*Apotomis betuletana*	B	linear	1	1635810
GCA_947179385	Lepidoptera	*Aricia agestis*	B	linear	2	1562496
GCA_947179375	Lepidoptera	*Carcina quercana*	B	linear	4	1572160
GCA_947179475	Lepidoptera	*Cydia splendana*	A	linear	2	1446399
GCA_947179575	Lepidoptera	*Erebia ligea*	B	linear	4	1520330
GCA_947250545	Lepidoptera	*Erynnis tages*	B	linear	1	1532960
GCA_947179535	Lepidoptera	*Idaea aversata*	B	linear	2	1828659
GCA_947179455	Lepidoptera	*Leptidea sinapis*	B	linear	4	1438040
GCA_947179425	Lepidoptera	*Ochlodes sylvanus*	B	linear	2	1713863
GCA_947179145	Lepidoptera	*Pandemis cinnamomeana*	B	linear	4	1779235
GCA_947251625	Lepidoptera	*Phalera bucephala*	B	linear	1	1358027
GCA_947251535	Lepidoptera	*Phalera bucephala*	B	linear	1	1462983
GCA_947250745	Lepidoptera	*Polyommatus icarus*	B	linear	1	1574132
GCA_947251505	Lepidoptera	*Thymelicus sylvestris*	B	linear	1	1286255
GCA_947251575	Lepidoptera	*Yponomeuta plumbellus*	A	linear	1	1418562
GCA_947251585	Odonata	*Ischnura elegans*	B	circular	1	2192296
GCA_947250715	Odonata	*Sympetrum striolatum*	A	linear	1	1516595
GCA_947251815	Orthoptera	*Chorthippus parallelus*	B	circular	1	1748465
GCA_947179555	Orthoptera	*Chorthippus brunneus*	B	linear	4	2013544
GCA_902646255.1	Trombidiformes	*Tetranychus urticae*	B	linear	149	1157560
GCA_019061405.1	Trombidiformes	*Fragariocoptes setiger*	E	linear	30	1082514
GCF_013309895.1	Pseudoscorpiones	*Atemnus politus*	S	linear	373	1445964
GCA_902636475.1	Coleoptera	*Diabrotica virgifera*	A	linear	105	1269619
GCF_020405475.1	Coleoptera	*Tribolium confusum*	B	linear	12	1418452
GCF_001931755.2	Entomobryomorpha	*Folsomia candida*	E	circular	1	1801626
GCF_017896245.1	Diptera	*Aedes aegypti*	A	circular	1	1267786
GCA_021609905.1	Diptera	*Delia radicum*	A	circular	1	1586584
GCF_008033215.1	Diptera	*Drosophila ananassae*	A	circular	1	1401460
GCF_001758565.1	Diptera	*Drosophila incompta*	A	circular	1	1267840
GCF_016584355.1	Diptera	*Drosophila melanogaster*	A	circular	1	1330657
GCF_014107455.1	Diptera	*Drosophila nikananu*	A	circular	1	1139744
GCF_018467135.1	Diptera	*Drosophila santomea*	A	circular	1	1409094
GCF_000022285.1	Diptera	*Drosophila simulans*	A	circular	1	1445873
GCF_014107475.1	Diptera	*Drosophila sturtevanti*	A	circular	1	1185354
GCA_009732755.1	Diptera	*Haematobia irritans*	A	circular	1	1352354
GCF_017604245.1	Diptera	*Rhagoletis cingulata*	A	circular	1	1538351
GCF_004171285.1	Diptera	*Aedes albopictus*	B	circular	1	1484007
GCF_018491625.2	Diptera	*Anopheles moucheti*	B	circular	1	1121812
GCF_008245065.1	Diptera	*Chrysomya megacephala*	B	circular	1	1376868
GCF_000073005.1	Diptera	*Culex quinquefasciatus*	B	circular	1	1482455
GCF_004795975.1	Diptera	*Drosophila mauritiana*	B	circular	1	1273530
GCF_000376585.1	Diptera	*Drosophila simulans*	B	circular	1	1301823
GCF_018454455.1	Diptera	*Ceratitis capitata*	A	linear	65	1239646
GCA_014129655.1	Diptera	*Drosophila arawakana*	A	linear	10	1293235
GCF_017916175.1	Diptera	*Drosophila auraria*	A	linear	116	1319217
GCF_014129605.1	Diptera	*Drosophila baimaii*	A	linear	26	1190958
GCF_014129645.1	Diptera	*Drosophila bicornuta*	A	linear	24	1182871
GCF_014129685.1	Diptera	*Drosophila bifasciata*	A	linear	17	1190237
GCF_014129615.1	Diptera	*Drosophila borealis*	A	linear	16	1214204
GCF_014129535.1	Diptera	*Drosophila neotestacea*	A	linear	19	1355678
GCF_014129565.1	Diptera	*Drosophila orientacea*	A	linear	19	1363981
GCA_000742435.1	Diptera	*Drosophila recens*	A	linear	43	1126656
GCA_014354315.1	Diptera	*Drosophila sechellia*	A	linear	83	1294885
GCA_002300525.1	Diptera	*Drosophila subpulchrella*	A	linear	106	1420341
GCA_000333795.2	Diptera	*Drosophila suzukii*	A	linear	110	1415350
GCA_005862135.1	Diptera	*Drosophila teissieri*	A	linear	122	1301330
GCF_014129515.1	Diptera	*Drosophila triauraria*	A	linear	9	1289544
GCF_014129525.1	Diptera	*Drosophila tropicalis*	A	linear	13	1215752
GCF_018454475.1	Diptera	*Rhagoletis cerasi*	A	linear	16	1255676
GCF_018491735.1	Diptera	*Anopheles demeilloni*	B	linear	64	1232500
GCA_000723225.2	Diptera	*Culex molestus*	B	linear	103	1435676
GCF_018454445.1	Diptera	*Rhagoletis cerasi*	B	linear	57	1180723
GCF_003999585.1	Hemiptera	*Bemisia tabaci*	B	circular	1	1306495
GCF_013458815.1	Hemiptera	*Diaphorina citri*	B	circular	1	1656288
GCF_000829315.1	Hemiptera	*Cimex lectularius*	F	circular	1	1250060
GCA_018224395.1	Hemiptera	*Homalodisca vitripennis*	B	linear	1	1712771
GCF_007115015.1	Hemiptera	*Laodelphax striatellus*	B	linear	2	1786382
GCF_007115045.1	Hemiptera	*Nilaparvata lugens*	B	linear	2	1542238
GCF_014534705.1	Hemiptera	*Pentalonia nigronervosa*	E	linear	182	1457187
GCA_017869155.1	Hymenoptera	*Ceratosolen solmsi*	A	circular	1	1206967
GCA_017869285.1	Hymenoptera	*Wiebesia pumilae*	A	circular	1	1278697
GCF_001439985.1	Hymenoptera	*Trichogramma pretiosum*	B	circular	1	1133809
GCA_014333535.1	Hymenoptera	*Anoplolepis gracilipes*	A	linear	96	1202684
GCF_010820705.1	Hymenoptera	*Apterostigma dentigerum*	A	linear	49	1174448
GCF_902713635.1	Hymenoptera	*Cardiocondyla obscurior*	A	linear	148	1194983
GCA_902648475.2	Hymenoptera	*Diachasma alloeum*	A	linear	169	1249264
GCA_902636375.1	Hymenoptera	*Dufourea novaeangliae*	A	linear	221	1188140
GCA_902636315.1	Hymenoptera	*Lasioglossum albipes*	A	linear	228	1195679
GCF_009012935.1	Hymenoptera	*Nasonia oneida*	A	linear	47	1293406

GCA_001983615.1	Hymenoptera	*Nasonia vitripennis*	A	linear	136	1198879
GCA_001675785.1	Hymenoptera	*Nomada ferruginata*	A	linear	231	1337641
GCF_001675695.1	Hymenoptera	*Nomada flava*	A	linear	167	1332780
GCA_001675715.1	Hymenoptera	*Nomada leucophthalma*	A	linear	182	1366719
GCA_001675775.1	Hymenoptera	*Nomada panzeri*	A	linear	191	1344355
GCF_006334525.1	Hymenoptera	*Leptopilina clavipes*	B	linear	46	1150755
GCA_902636395.1	Hymenoptera	*Osmia caerulescens*	F	linear	163	1232261
GCF_006542295.1	Lepidoptera	*Carposina sasakii*	A	circular	1	1449344
GCF_020995475.1	Lepidoptera	*Corcyra cephalonica*	B	circular	3	1359904
GCF_018141665.1	Lepidoptera	*Spodoptera picta*	B	circular	1	1339720
GCF_018555315.1	Lepidoptera	*Erebia cassioides*	B	linear	2	1423447
GCF_000333775.1	Lepidoptera	*Hypolimnas bolina*	B	linear	144	1377933
GCF_001266585.1	Lepidoptera	*Operophtera brumata*	B	linear	120	1121046
GCA_002318985.1	Lepidoptera	*Plutella australiana*	B	linear	95	1158805
GCF_013365455.1	Nematoda	*Dirofilaria immitis*	C	circular	1	920122
GCF_000306885.1	Nematoda	*Onchocerca ochengi*	C	circular	1	957990
GCF_000008385.1	Nematoda	*Brugia malayi*	D	circular	1	1080084
GCF_012030695.1	Nematoda	*Brugia pahangi*	D	circular	1	1072967
GCF_013365435.1	Nematoda	*Litomosoides sigmodontis*	D	circular	1	1045802
GCF_013365475.1	Nematoda	*Cruorifilaria tuberocauda*	J	circular	1	863988
GCF_013365495.1	Nematoda	*Dipetalonema caudispina*	J	circular	1	863427
GCF_000530755.1	Nematoda	*Onchocerca volvulus*	C	linear	1	960618
GCF_013366805.1	Nematoda	*Litomosoides brasiliensis*	D	linear	41	1046149
GCF_002204235.2	Nematoda	*Wuchereria bancrofti*	D	linear	100	1060850
GCA_001752665.1	Nematoda	*Pratylenchus penetrans*	E	linear	12	975127
GCF_013366855.1	Nematoda	*Madathamugadia hiepei*	F	linear	208	1025329
GCF_020278625.1	Nematoda	*Mansonella ozzardi*	F	linear	93	1073310
GCF_012277295.1	Siphonaptera	*Ctenocephalides felis*	E	circular	1	1495538
GCF_012277315.1	Siphonaptera	*Ctenocephalides felis*	F	circular	1	1201647
GCA_902636825.1	Lepidoptera	*Polygonia c-album*	B	linear	294	1300744
GCF_032584155.1	Diptera	*Drosophila bocqueti*	A	linear	255	1134576
GCF_000689175.1	Diptera	*Glossina morsitans morsitans*	A	linear	201	1019510
GCF_028982005.1	Diptera	*Drosophila bocki*	A	linear	227	1090928
GCF_028981785.1	Diptera	*Drosophila leontia*	A	linear	186	1096851
GCF_032584075.1	Diptera	*Drosophila seguyi*	A	linear	197	1167524
GCA_023661065.1	Diptera	*Melophagus ovinus*	F	linear	196	1008858
GCF_020278605.1	Nematoda	*Mansonella perstans*	F	linear	170	1058123
GCA_033843885.1	Hymenoptera	*Andrena praecox*	A	linear	187	1279697
GCA_033844085.1	Hymenoptera	*Andrena apicata*	A	linear	168	1133707
GCF_028981925.1	Diptera	*Drosophila burlai*	A	linear	128	1248003
GCA_902647005.1	Diptera	*Sphyracephala brevicornis*	A	linear	160	1235914
GCF_033042245.1	Diptera	*Zaprionus taronus*	A	linear	136	1390699
GCA_029784575.1	Phthiraptera	*Penenirmus auritus*	F	linear	140	1198730
GCF_032584135.1	Diptera	*Drosophila tristis*	A	linear	116	1267248
GCA_033843805.1	Hymenoptera	*Lasioglossum nitidulum*	A	linear	116	1206526
GCA_033843905.1	Hymenoptera	*Andrena minutula*	A	linear	113	1198381
GCA_033844045.1	Hymenoptera	*Andrena bicolor*	A	linear	110	1265543
GCF_032584115.1	Diptera	*Scaptomyza pallida*	A	linear	90	1309055
GCA_026768255.1	Hymenoptera	*Acromyrmex echinatior*	A	linear	77	1522719
GCF_028982115.1	Diptera	*Drosophila tsacasi*	A	linear	59	1132109
GCF_014771645.1	Hemiptera	*Cimex hemipterus*	U	linear	1	1291339
GCF_029169405.1	Nematoda	*Onchocerca gibsoni*	C	linear	1	997610
GCA_030441635.1	Sarcoptiformes	*Tyrophagus putrescentiae*	E	linear	25	1028159
GCA_029784595.1	Phthiraptera	*Meromenopon meropis*	F	linear	9	1005754
GCF_023559125.1	Lepidoptera	*Ostrinia furnacalis*	B	circular	1	1321828
GCF_023559145.1	Lepidoptera	*Ostrinia scapulalis*	B	circular	1	1320340
GCF_024205405.1	Trombidiformes	*Tetranychus truncatus*	B	linear	1	1515617
GCF_025617515.1	Coleoptera	*Oryzaephilus surinamensis*	B	circular	1	1728764
GCF_029856955.1	Thysanoptera	*Frankliniella intonsa*	B	circular	1	1463884
GCA_963931955.1	Coleoptera	*Longitarsus flavicornis*	A	circular	1	1627592
GCA_963969245.1	Hymenoptera	*Ophion costatus*	A	circular	1	1399493
GCF_032848195.1	Diptera	*Drosophila aff. chauvacae*	A	circular	1	1244713
GCA_037076435.1	Diptera	*Drosophila biauraria*	A	circular	1	1178058
GCF_021378375.1	Diptera	*Drosophila innubila*	A	circular	1	1290587
GCA_963966195.1	Hymenoptera	*Nomada hirtipes*	A	circular	1	1442356
GCF_918697765.1	Coleoptera	*Phyllotreta cruciferae*	A	circular	1	1795892
GCA_028571345.1	Siphonaptera	*Ctenocephalides orientis*	E	circular	1	1371830
GCA_023052945.1	Hymenoptera	*Camponotus pennsylvanicus*	A	circular	1	1582319
GCF_918342435.1	Coleoptera	*Psylliodes chrysocephala*	A	circular	1	1438854
GCF_051130665	Coleoptera	*Zygogramma bicolorata*	A	circular	1	1408155
GCA_018467115	Diptera	*Drosophila yakuba*	A	circular	1	1394392
GCA_041439025	Isopoda	*Armadillidium vulgare*	B	circular	1	1711483
GCA_000376605	Diptera	*Drosophila simulans*	A	circular	1	1295804
GCA_045865035	Lepidoptera	*Eurema mandarina*	B	circular	1	1358360
GCA_045865025	Lepidoptera	*Ephestia kuehniella*	A	circular	1	1305037
GCA_045865015	Lepidoptera	*Cadra cautella*	B	circular	1	1703391
GCA_026015925	Diptera	*Drosophila pseudotakahashii*	A	circular	1	1308415
GCA_041442225	Isopoda	*Armadillidium vulgare*	B	circular	1	1566000
GCA_041440575	Isopoda	*Armadillidium vulgare*	B	circular	1	1638198
GCA_000008025	Diptera	*Drosophila melanogaster*	A	circular	1	1267782
GCA_037076445	Diptera	*Drosophila biauraria*	A	circular	1	1183391
GCA_030295095	Lepidoptera	*Homona magnanima*	B	circular	1	1542158
GCA_026274225	Diptera	*Drosophila pseudotakahashii*	A	linear	127	1564190
GCA_045865005	Lepidoptera	*Cadra cautella*	A	circular	1	1713404
GCA_040084705	Diptera	*Drosophila willistoni*	A	circular	1	1268528

Computational code used in data analysis.

1. Retrieve selected genomes from NCBI

```
dos2unix accession_list.txt
while read accession; do
  datasets download genome accession $accession --include genome
  unzip -q -o ncbi_dataset.zip \
           "ncbi_dataset/data/$accession*/$accession*.fna"
  mv ncbi_dataset/data/$accession*/$accession*.fna ./genomes/$accession
  rm -f -r ncbi_dataset*
done < accession_list.txt
```

2. Retrieve protein sequences of coding regions using Prokka

```
for file in ./wolb251/*.fasta; do
      i=$(basename ${file%.*})
      prokka --outdir ./wolb251_proteome/$i --prefix $i --locustag $I \
      --genus wolbachia --kingdom Bacteria --noanno --cpus 20 \
      wolb251/$i.fasta;
done
```

3. BUSCO analysis of genomes

```
for file in ./wolb251_proteome/*.faa; do
      name=$(basename ${file%.*})
      busco -i $file -m proteins -c 40 -l rickettsiales_odb12 --out $name \
      --out_path busco_ricket --offline --download_path busco_db;
done
```

4. Phylogeny based on BUSCO markers

```
BUSCO_phylogenomics.py -i busco_ricket -o busco_phylo -t 40 \
   --supermatrix_only --percent_single_copy 95
iqtree2 -s SUPERMATRIX.phylip -m LG+G4 -T 40 -B 1000 -alrt 1000 \
   --prefix OG323
```

5. MMSeqs2 based homology search

```
cat ./wolb251_proteome/*.faa >> wolb251_proteome.fasta
cat wolb251_proteome.fasta | seqkit replace -p "\s.+" > wolb251_proteomi.fa
mmseqs easy-search reference_WMK.fasta wolb251_proteomi.fa wmk-homologs.out \
   tmp --min-seq-id 0.40 --format-mode 4 --max-seqs 1500 --format-output \
   "query,target,fident,alnlen,mismatch,gapopen,qstart,qend,tstart,tend, \
   Evalue, bits,tlen,qcov"

>> reference_WMK.fasta includes three sequences wBic-Wmk, wMel-Wmk and wZbi-
Wmk
```

6. Homology filtering

```
awk 'NR==1 && /^query[[:space:]]/ {print; next} $1=="QEQ51101_wBif" && $7<30\
   && $8>199 {print}' wmk-homologs.out > wBifWmk_homologs_2HTH.tsv
awk 'NR==1 && /^query[[:space:]]/ {print; next} $1=="XXB89297_wZbi" && $7<22\
   && $8>191 {print}' wmk-homologs.out > wZbiWmk_homologs_2HTH.tsv
awk 'NR==1 && /^query[[:space:]]/ {print; next} $1=="AAS14326_WD0626_wMel"\
   && $7<15 && $8>220 {print}' wmk-homologs.out > wMelWmk_homologs_2HTH.tsv
```

```
awk 'FNR==1 && NR!=1 {next} {print}' wBifWmk_homologs_2HTH.tsv \
   wZbiWmk_homologs_2HTH.tsv wMelWmk_homologs_2HTH.tsv \
   > allWmk_homologs_2HTH.tsv
awk 'NR>1 {print $2}' allWmk_homologs_2HTH.tsv | sort | uniq \
   > uniqueWmk_homologs_2HTH.id
seqkit grep -f uniqueWmk_homologs_2HTH.id wolb251_proteomi.fasta \
   > uniqueWmk_homologs_2HTH.fasta
```

7. Wmk phylogenetic tree reconstruction

```
mafft --auto --thread 8 uniqueWmk_homologs_2HTH.fasta > wmk1205_aln.fasta
trimal -automated1 -in wmk1205_aln.fasta -out wmk1205_aln_trm.fasta
iqtree2 -s wmk1205_aln_trm.fasta -m MFP -T AUTO -B 1000 -alrt 1000 \
   --prefix wmk1205
```

8. Bayesian tree analysis

```
seqmagick convert --alphabet protein wmk1205_aln_trm.fasta wmk1205.nex
begin mrbayes;
   lset rates=gamma;
   prset aamodelpr=mixed;
   mcmc ngen=5000000 nchains=4 nruns=2 samplefreq=500 printfreq=1000
savebrlens=yes filename=wmk1205;
   sump;
   sumt;
end;
mpiexec -np 16 mb -i wmk1205.nex
```

9. Phylogenetic tree with marker sequences from previous study

```
cat uniqueWmk_homologs_2HTH.fasta lefoulon_wmkhomologs.fasta >> wmk1314.fasta
mafft --auto --thread 8 wmk1314.fasta > wmk1314_aln.fasta
trimal -automated1 -in wmk1314_aln.fasta -out wmk1314_aln_trm.fasta
iqtree2 -s wmk1314_aln_trm.fasta -m MFP -T AUTO -B 1000
```

10. Phylogenetic tree after prunning aligned regions encompassing Type VI specific deletions

```
seqkit subseq -r 1:98 wmk1205_aln_trm.fasta > tmp1.fasta && \
seqkit subseq -r 146:-1 wmk1205_aln_trm.fasta > tmp2.fasta && \
seqkit concat tmp1.fasta tmp2.fasta > wmk1205_aln_trm_gl.fasta && \
rm tmp1.fasta tmp2.fasta
iqtree2 -s wmk1205_aln_trm_gl.fasta -m Q.bird+F+R6 -T AUTO -B 1000 -alrt 1000
   --prefix wmk1205gl
```

11. Multiple sequence alignment of homologs

```
cat ./canonicalWmk/*.fasta >> canonicalWmk.fasta
cat ./distantWmk/*.fasta >> distantWmk.fasta
cat canonicalWmk.fasta distantWmk.fasta >> allWmk.fasta
mafft --auto --thread 8 allWmk.fasta > allWmk_aln.fasta
trimal -automated1 -in allWmk_aln.fasta -out allWmk_aln_trm.fasta

seqkit sample -n 35 -s 53618 canonicalWmk.fasta -o canonicalWmk_30.fasta
seqkit sample -n 20 -s 53618 distantWmk.fasta -o distantWmk_20.fasta
cat canonicalWmk_30.fasta distantWmk_20.fasta >> allWmk_50.fasta
>> Alignment using Uniprot online server
```

12. Assigning phylo-typing to FlaG-analysed sequences

```
cat wmk1205.fasta wmk146_webflagrun1.fasta wmk2_webflagrun2.fasta \
   wmk32_webflagrun3.fasta  >> wmk1385.fasta
mafft --auto --thread 8 wmk1385.fasta > wmk1385_aln.fasta
trimal -automated1 -keepheader -in wmk1385_aln.fasta -out \
   wmk1385_aln_trm.fasta
iqtree2 -g wmk1205.treefile -s wmk1385_aln_trm.fasta -m Q.bird+F+R6 -T AUTO \
   -pre wmk1385
```

13. Protein structure prediction for Wmk homologs

```
>> all homologs of wZbi-wmk and wBif-wmk and sampled homologs of wmel-wmk
>> protein structure prediction with AlphaFold2 server using ColabFold
```

14. Structure based phylogeny using 166 top rank AF2 conformations

```
foldseek createdb ./pdb166/ db
foldseek convert2fasta db pdb166_aa.fasta
foldseek lndb db_h db_ss_h
foldseek convert2fasta db_ss pdb166_3di.fasta
cp mat3di.out Q.3Di.AF.tsv Q.3Di.LLM.tsv
mafft --globalpair --maxiterate 1000 pdb166_aa.fasta > pdb166_aa_aln.fasta
mafft --globalpair --maxiterate 1000 --aamatrix mat3di.out pdb166_3di.fasta \
   > pdb166_3di_aln.fasta
trimal -automated1 -in pdb166_aa_aln.fasta -out pdb166_aa_aln_trm.fasta
trimal -automated1 -in pdb166_3di_aln.fasta -out pdb166_3di_aln_trm.fasta
seqkit concat pdb166_aa_aln_trm.fasta pdb166_3di_aln_trm.fasta \
   >> pdb166_aa3di_merged.fasta
iqtree2 -s pdb166_3di_aln_trm.fasta -m MF  \
   -mset GTR20,Q.3Di.AF.tsv,Q.3Di.LLM.tsv -mrate G -pre modeltest

iqtree2 -s pdb166_aa_aln_trm.fasta -m LG+F+G4 -T AUTO -B 1000 -alrt 1000 \
   --prefix model1
iqtree2 -s pdb166_aa3di_merged.fasta -p partitions_1.nex -mdef 3DIPHY.nexus \
   -T AUTO -B 1000 -alrt 1000 --prefix model2
iqtree2 -s pdb166_aa3di_merged.fasta -p partitions_2.nex -mdef Q3DIAF.nexus \
   -T AUTO -B 1000 -alrt 1000 --prefix model3

>> FoldTree based analysis used online server through colab notebook
```

15. Structure comparison using FoldSeek 3Di+AA TMscore (166 homologs)

```
foldseek easy-search ./pdb166 ./pdb166 pdb166_foldseek3A.tsv tmp  \
   --exhaustive-search 1 --alignment-type 2 --exact-tmscore 1 \
   --format-output query,target,alnlen,qcov,tcov,pident,lddt,alntmscore, \
   qtmscore,ttmscore,u,t,rmsd,evalue,prob
```

16. Structure comparison using FATCAT (166 homologs)

```
ls ./pdb166/*.pdb | sed 's/\.pdb$//' | sort | awk '{a[NR]=$0} \
   END{for(i=1;i<=NR;i++) for(j=i+1;j<=NR;j++) print a[i], a[j]}' \
   >> pdb166_fatcat_pairs.txt

FATCATQue.pl timeused pdb166_fatcat_pairs.txt -q > pdb166_fatcat_aln.out
```
