# Supplementary material for: Structurally reorganized Wmk lineage with distinct genomic context and restricted distribution in Wolbachia
Source: G3 (Bethesda). 2026 Aug 27;16(11):jkag234. doi: 10.1093/g3journal/jkag234 (PMC13732484; doi:10.1093/g3journal/jkag234)
Supplement: Supplementary file 1 [file jkag234_supplementary_data.zip › Supplemental_Material_G3-2026-407105.pdf]

Supplementary file for the title: Structurally reorganized Wmk lineage with distinct genomic context and restricted distribution in *Wolbachia*

Table S1. Comparative summary of full-length Wmk homolog counts in 30 *Wolbachia* strains, contrasting the numbers detected in the present study with those reported in a previous investigation.

**Strain Name**	**Genome accession**	**GenBank accession**	**Count of 2HTH-Wmk in current study**	**Count of 2HTH-Wmk in Lefoulon et al 2025**
*w*Ana	GCA_008033215	CP042904	3	3
*w*Biau1	GCA_037076435	AP028655	0	0
*w*Biau2	GCA_037076445	AP028656	0	1
*w*Bif	GCA_014129685	JAATLC010000010	1	2
*w*Bol1	GCA_000333775	CAOH01000062	5	5
*w*Bor	GCA_014129615	JAATLD010000002	3	3
*w*CauA	GCA_045865005	AP028948	14	17
*w*CauB	GCA_045865015	AP028949	11	12
*w*CI	GCA_045865035	AP028951	4	6
*w*Fur	GCA_023559125	CP096925	6	7
*w*Ha	GCA_000376605	CP003884	3	3
*w*Hm-t	GCA_030295095	AP025638	7	8
*w*Inn	GCA_021378375	CP076228	3	3
*w*Kue	GCA_045865025	AP028950	2	2
*w*Mau	GCA_004795975	CP034335	1	2
*w*Mel	GCA_000008025	AE017196	4	5
*w*No	GCA_000376585	CP003883	1	1
*w*Pip	GCA_000073005	NC_010981	4	4
*w*Pse-N101	GCA_026274225	JAPJVH010000002	3	4
*w*Pse-Smith	GCA_026015925	CP110359	3	4
*w*Rec	GCA_000742435	JQAM01000018	1	1
*w*Ri	GCA_000022285	CP001391	6	6
*w*Sca	GCF_023559145	CP096926	6	7
*w*Spc	GCA_002300525	NTHL01000073	3	3
*w*Tpre	GCA_001439985	CM003641	0	0
*w*VulC	GCA_041439025	CP156068	9	8
*w*VulM	GCA_041440575	CP156069	5	6
*w*VulP	GCA_041442225	CP156070	6	6
*w*Wil	GCA_040084705	CP157591	4	4
*w*Yak	GCA_018467115	CP069053	6	6

Table S2. Median structural similarity metrics among the six Wmk Types based on pairwise conformational comparisons. FATCAT scores are presented in the lower-left panel of the matrix, whereas TM-align TM-scores are shown in the upper-right panel. Cyan-colored cells denote comparisons involving Type VI against other Types.

	Type I	Type II	Type III	Type IV	Type V	Type VI
Type I		0.434 (55.00)	0.475 (42.00)	0.455 (42.90)	0.424 (37.10)	0.318 (27.20)
Type II	70.315 (3, 4.68)		0.495 (42.20)	0.513 (44.55)	0.434 (37.70)	0.278 (26.90)
Type III	58.88 (4, 7.17)	60 (3, 7.54)		0.864 (56.10)	0.487 (34.80)	0.290 (24.70)
Type IV	63.64 (3, 5.66)	62.26 (3, 5.98)	71.1 (0, 3.9)		0.493 (32.70)	0.270 (24.90)
Type V	55.35 (4, 7.42)	53.47 (3, 6.93)	50.65 (4, 9.52)	50.24 (3, 8.14)		0.292 (23.50)
Type VI	43.88 (3, 17.88)	43.84 (3, 17.82)	37.46 (4, 22.3)	38.44 (4, 22.07)	38.99 (3, 18.18)	

TM-score (pident)

FATCAT similarity (twists count, gaps percent)

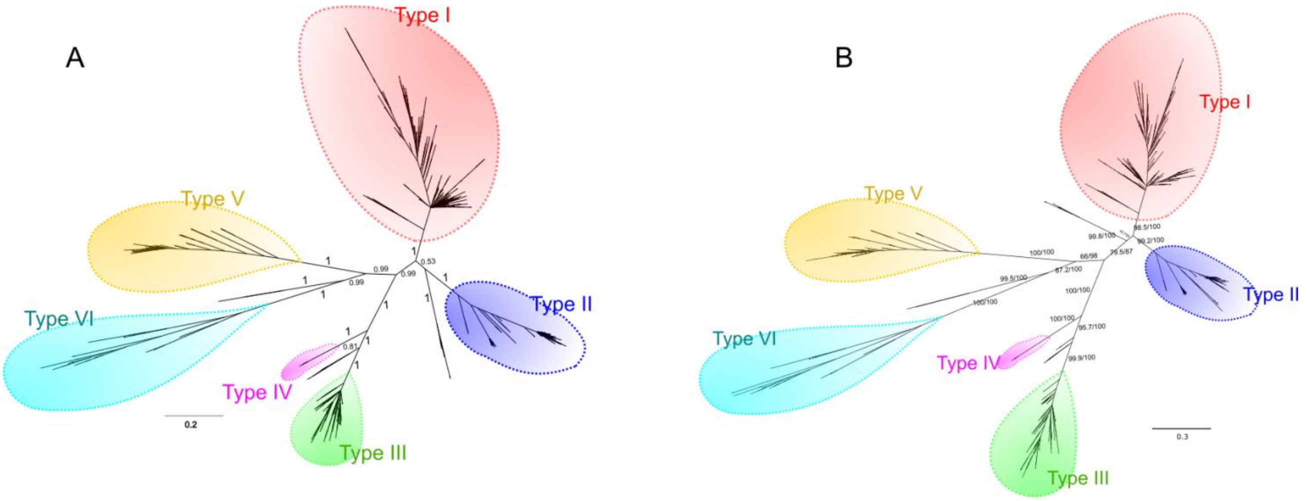

Figure S1. Phylogenetic reconstruction of Wmk homologs under alternative inference frameworks.
(A) Bayesian phylogeny of 1205 Wmk homologs identified from 207 *Wolbachia* genomes. Node support values are posterior probabilities and are shown for deeper nodes only. Major clades corresponding to Type I–VI groups are highlighted with colored balloons. Homologs not assigned to any of the six types are positioned outside the highlighted regions.
(B) Maximum likelihood phylogeny of the same 1205 Wmk homologs after pruning the alignment to remove positions 99–145, which encompass the characteristic deletion observed in Type VI relative to Type I–V. Node support values are shown as approximate likelihood ratio test and ultrafast bootstrap percentages (aLRT/BS) calculated from 1000 replicates. Major clades corresponding to Type I–VI groups are highlighted with colored balloons, and unclassified homologs are positioned outside these regions.

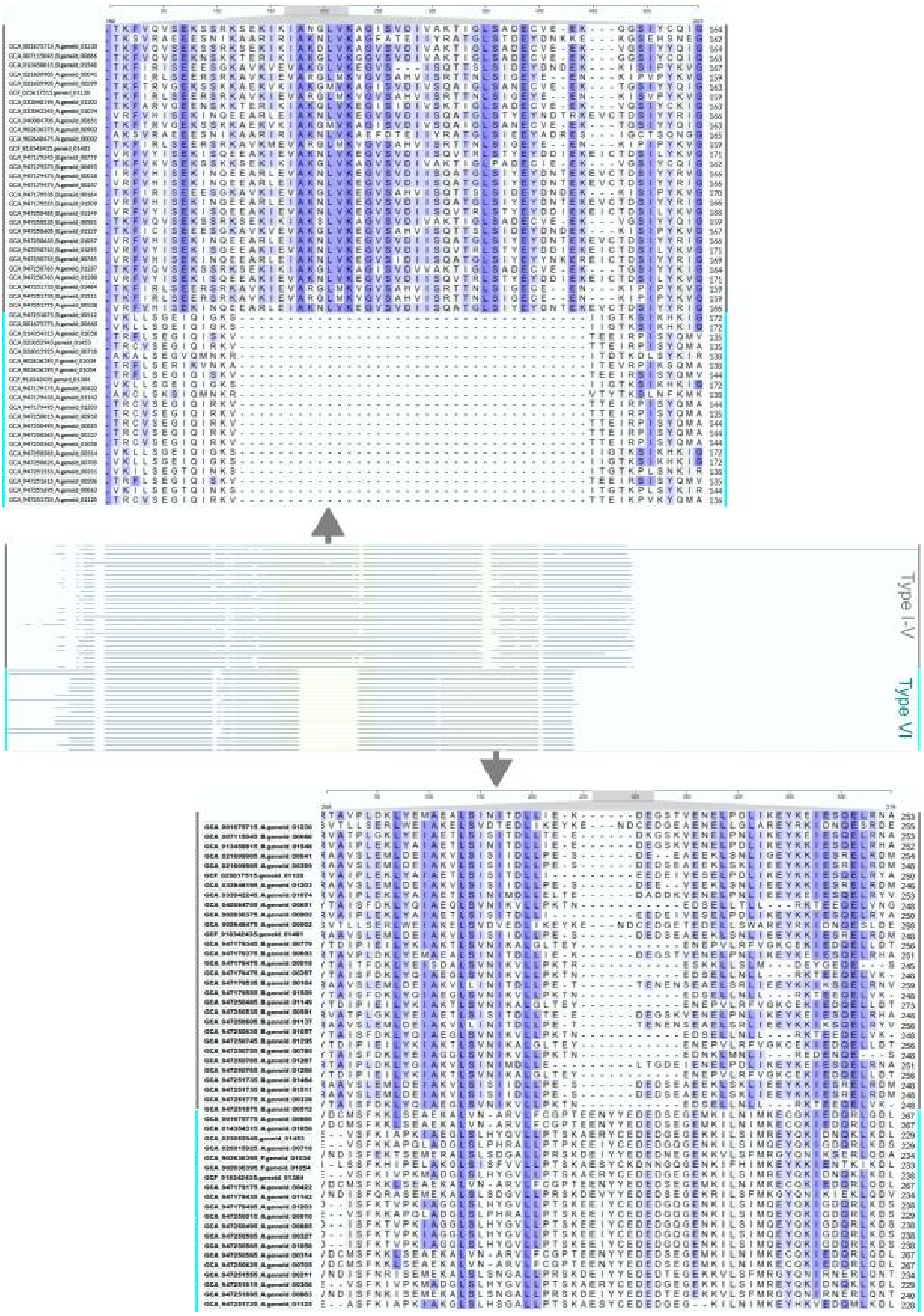

Figure S2. Snapshot of the multiple sequence alignment of 50 Wmk homologs highlighting the region encompassing the characteristic sequence deletion (upper panel) and insertion (lower panel) in Type VI homologs. The alignment was generated using Clustal Omega via the UniProt server and includes 30 Type I–V homologs (indicated by gray side bars) and 20 Type VI homologs (indicated by cyan side bars).

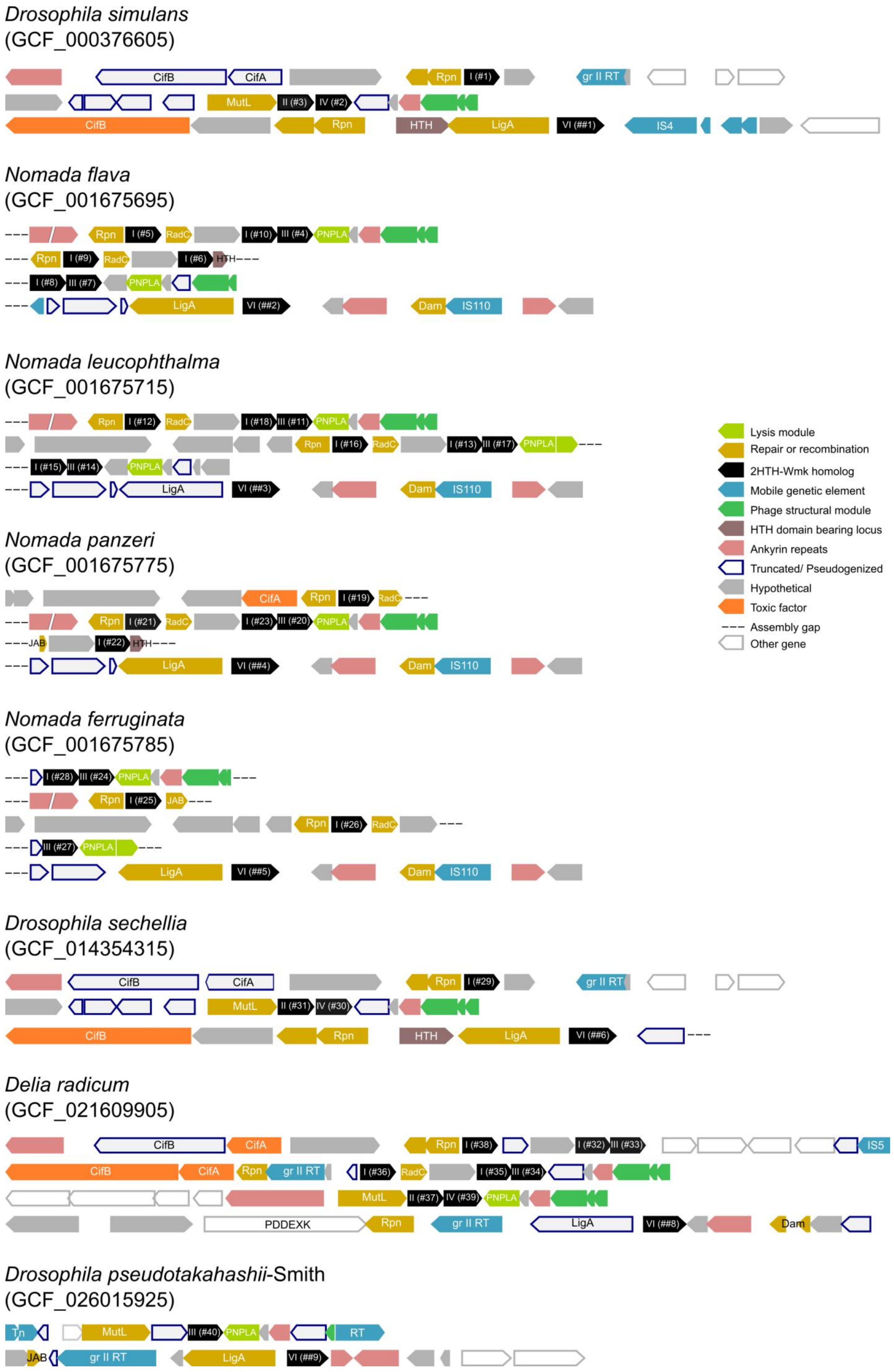

Drosophila simulans
(GCF_000376605)
CifB CifA Rpn I (#1) gr II RT
MutL II (#3) IV (#2)
CifB Rpn HTH LigA VI (##1) IS4
Nomada flava
(GCF_001675695)
Rpn I (#5) RadC I (#10) III (#4) PNPLA
Rpn I (#9) RadC I (#6) HTH
I (#8) III (#7) PNPLA
LigA VI (##2) Dam IS110
Nomada leucophthalma
(GCF_001675715)
Rpn I (#12) RadC I (#18) III (#11) PNPLA
Rpn I (#16) RadC I (#13) III (#17) PNPLA
I (#15) III (#14) PNPLA
LigA VI (##3) Dam IS110
Nomada panzeri
(GCF_001675775)
CifA Rpn I (#19) RadC
Rpn I (#21) RadC I (#23) III (#20) PNPLA
JAB I (#22) HTH
LigA VI (##4) Dam IS110
Nomada ferruginata
(GCF_001675785)
I (#28) III (#24) PNPLA
Rpn I (#25) JAB
Rpn I (#26) RadC
III (#27) PNPLA
LigA VI (##5) Dam IS110
Drosophila sechellia
(GCF_014354315)
CifB CifA Rpn I (#29) gr II RT
MutL II (#31) IV (#30)
CifB Rpn HTH LigA VI (##6)
Delia radicum
(GCF_021609905)
CifB CifA Rpn I (#38) I (#32) III (#33) IS5
CifB CifA Rpn gr II RT I (#36) RadC I (#35) III (#34)
MutL II (#37) IV (#39) PNPLA
PDDEXK Rpn gr II RT LigA VI (##8) Dam
Drosophila pseudotakahashii-Smith
(GCF_026015925)
Tn MutL III (#40) PNPLA RT
JAB gr II RT LigA VI (##9)
Lysis module
Repair or recombination
2HTH-Wmk homolog
Mobile genetic element
Phage structural module
HTH domain bearing locus
Ankyrin repeats
Truncated/ Pseudogenized
Hypothetical
Toxic factor
Assembly gap
Other gene

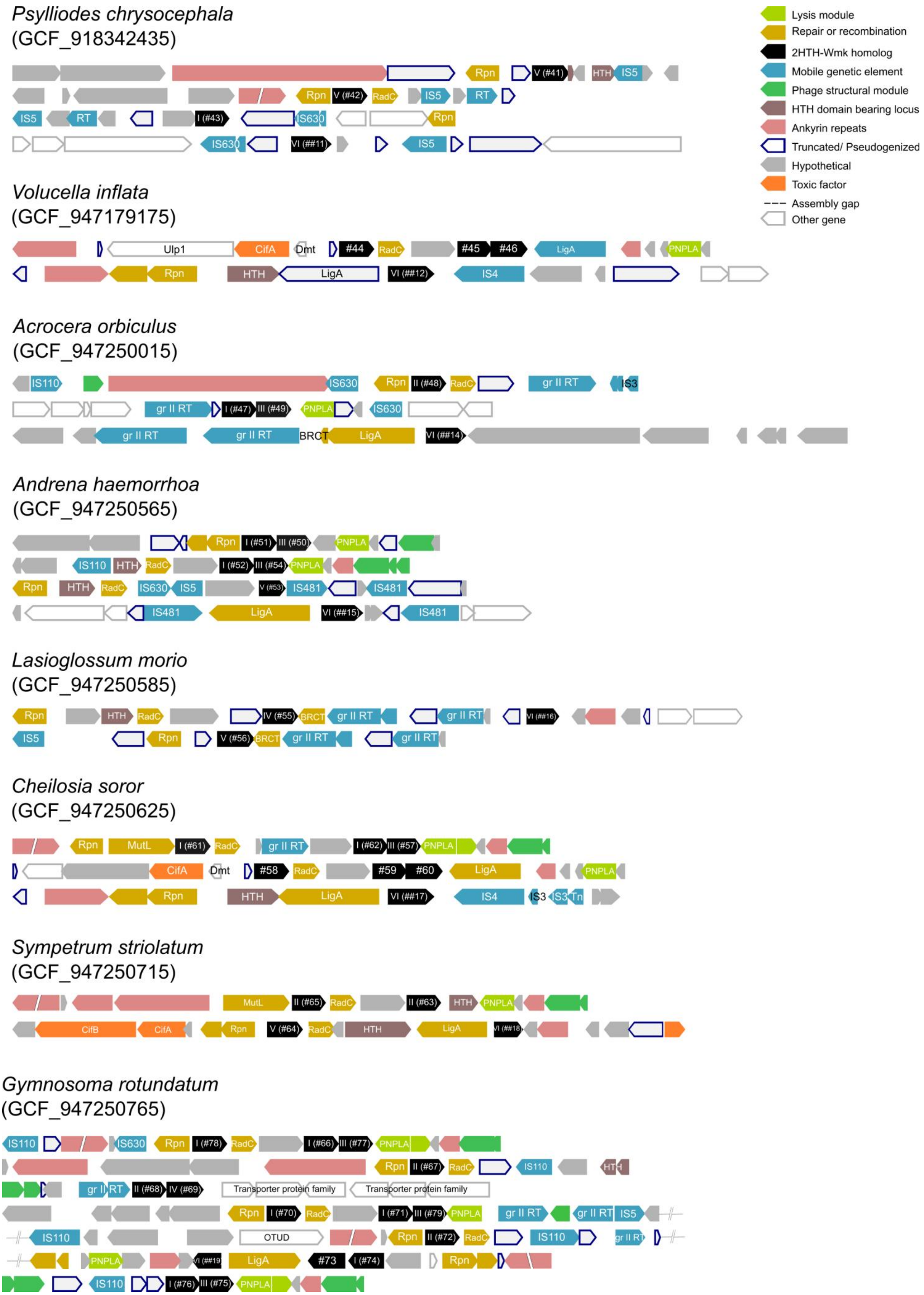
Psylliodes chrysocephala
(GCF_918342435)
Volucella inflata
(GCF_947179175)
Acrocera orbiculus
(GCF_947250015)
Andrena haemorrhoa
(GCF_947250565)
Lasioglossum morio
(GCF_947250585)
Cheilosia soror
(GCF_947250625)
Sympetrum striolatum
(GCF_947250715)
Gymnosoma rotundatum
(GCF_947250765)
Lysis module
Repair or recombination
2HTH-Wmk homolog
Mobile genetic element
Phage structural module
HTH domain bearing locus
Ankyrin repeats
Truncated/ Pseudogenized
Hypothetical
Toxic factor
Assembly gap
Other gene

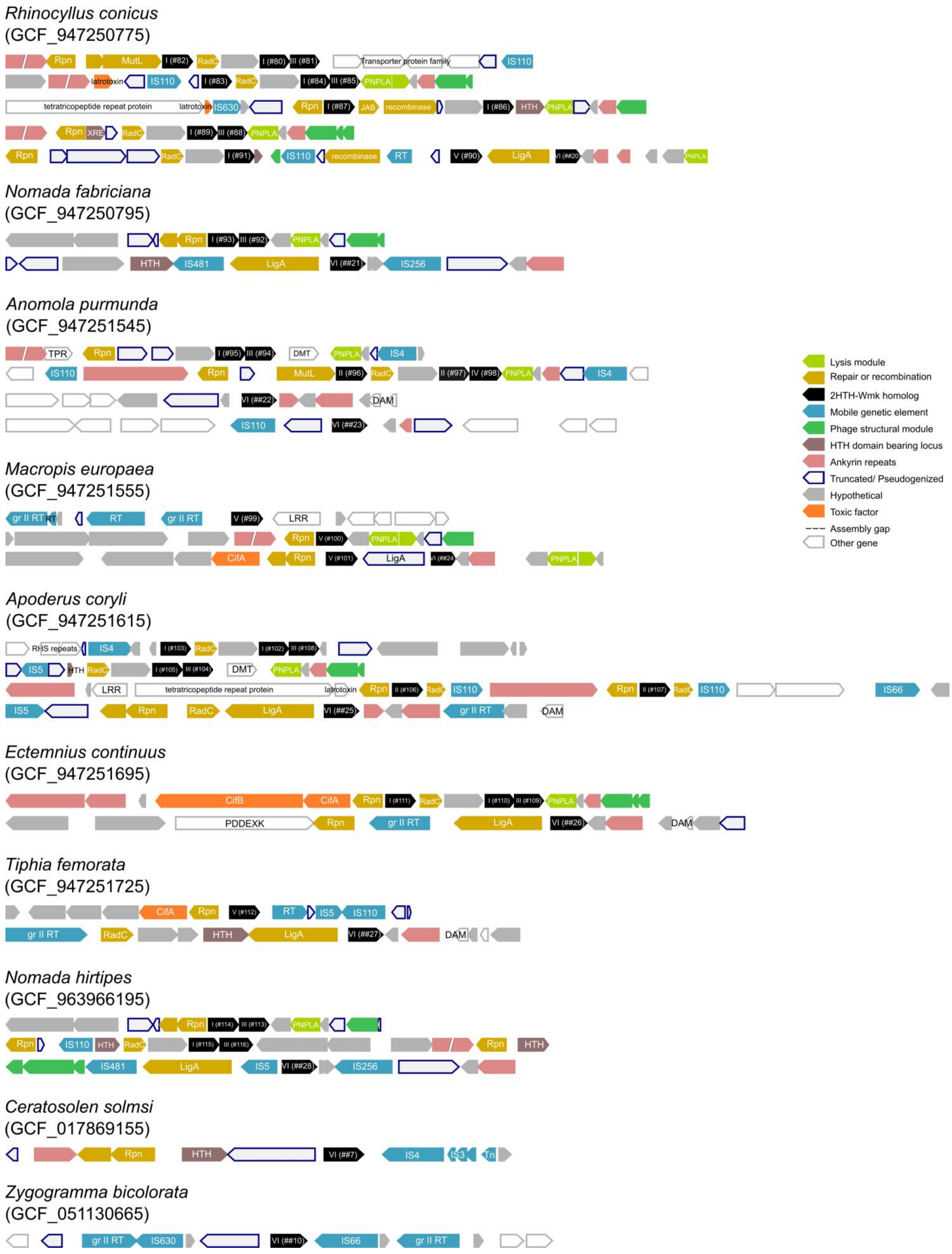
Rhinocyllus conicus
(GCF_947250775)
Nomada fabriciana
(GCF_947250795)
Anomola purmunda
(GCF_947251545)
Macropis europaea
(GCF_947251555)
Apoderus coryli
(GCF_947251615)
Ectemnius continuus
(GCF_947251695)
Tiphia femorata
(GCF_947251725)
Nomada hirtipes
(GCF_963966195)
Ceratosolen solmsi
(GCF_017869155)
Zygogramma bicolorata
(GCF_051130665)
Lysis module
Repair or recombination
2HTH-Wmk homolog
Mobile genetic element
Phage structural module
HTH domain bearing locus
Ankyrin repeats
Truncated/ Pseudogenized
Hypothetical
Toxic factor
Assembly gap
Other gene

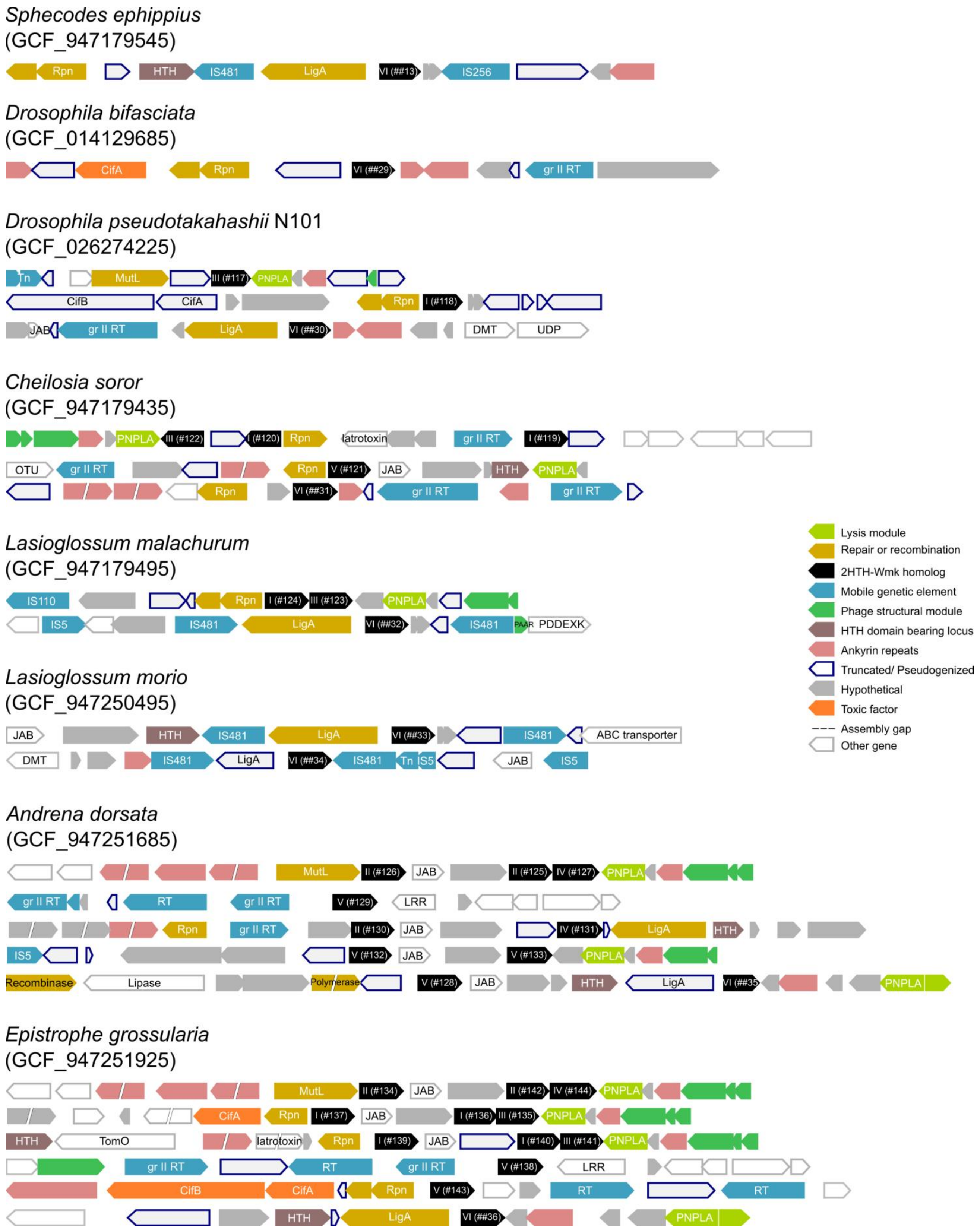

Figure S3. Genomic neighborhood architecture of Wmk homologs (black modules) in 34 selected *Wolbachia* strains. Flanking genes are shown to illustrate local synteny patterns. Gene map legends are provided on the right. Labels within the black Wmk homolog modules indicate Wmk type, while numbers in parentheses correspond to the reference numbers of the annotated loci in Supplementary Data 2.

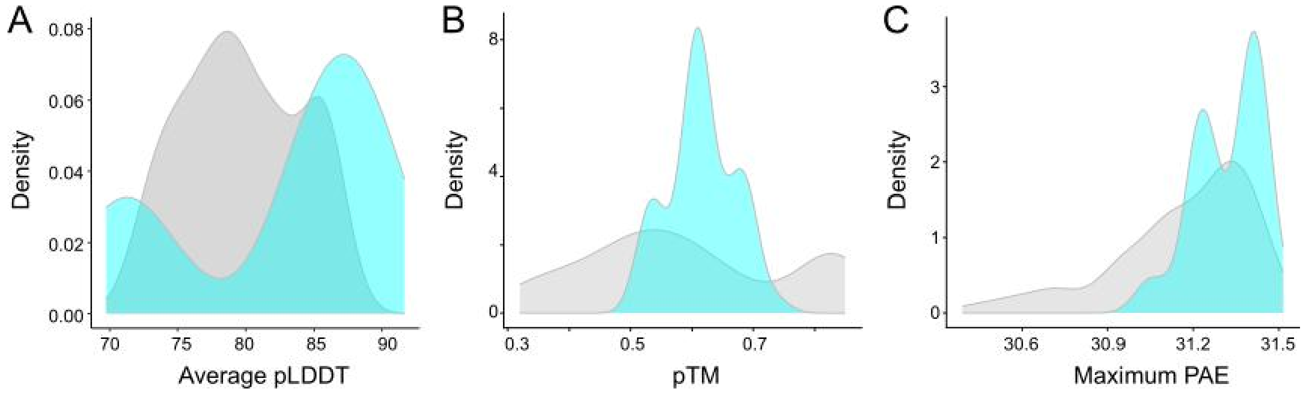

Figure S4. Distribution of model confidence metrics for AlphaFold-predicted structures of 166 Wmk homologs. Shown are the distributions of average pLDDT, predicted TM-score (pTM), and maximum predicted aligned error (max PAE). Values for Type VI homologs (52 structures) are indicated in cyan, whereas those for Type I–V homologs (114 structures) are shown in gray.

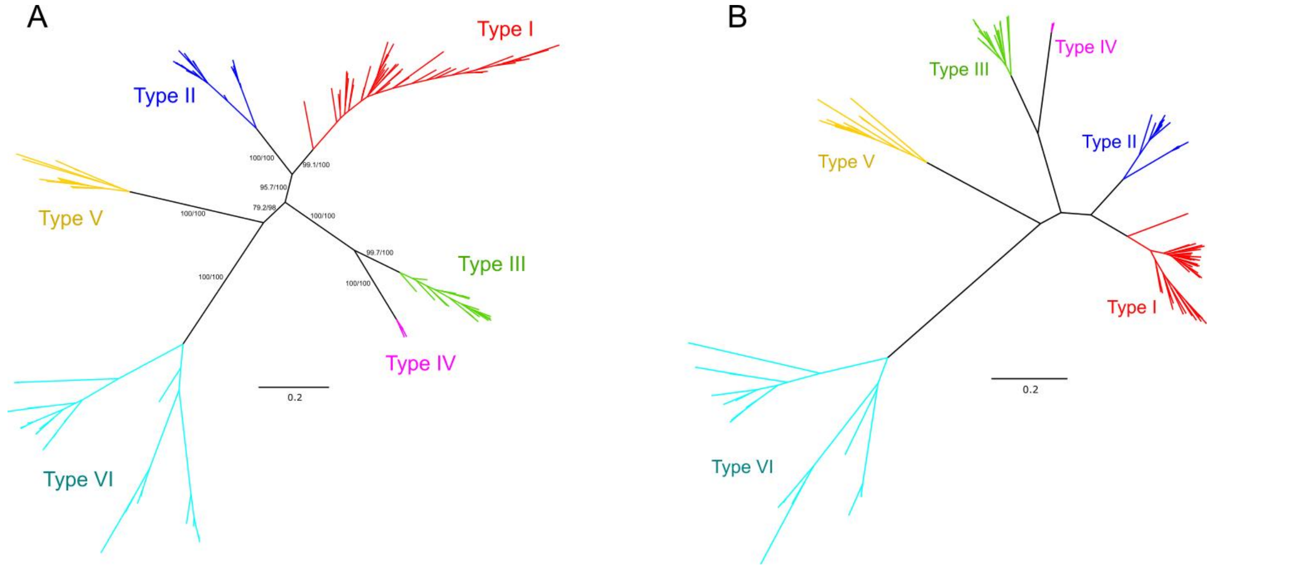

Figure S5: (A) Structure-informed maximum likelihood phylogeny of 166 Wmk homologs derived from 106 *Wolbachia* genomes. The tree was reconstructed using a partitioned framework in which protein sequence and structural alphabets were treated as separate partitions. The structural partition was modeled using 3DIPHY. Node supports (aLRT/BS) are based on 1000 replicates. Branches are colored according to the sequence-based Type classification shown in Fig. 2A and labeled accordingly.

(B) Phylogenetic tree inferred using FoldTree analysis of the same 166 structures. Branches are colored and labeled according to the sequence-based Type classification defined in Fig. 2A.

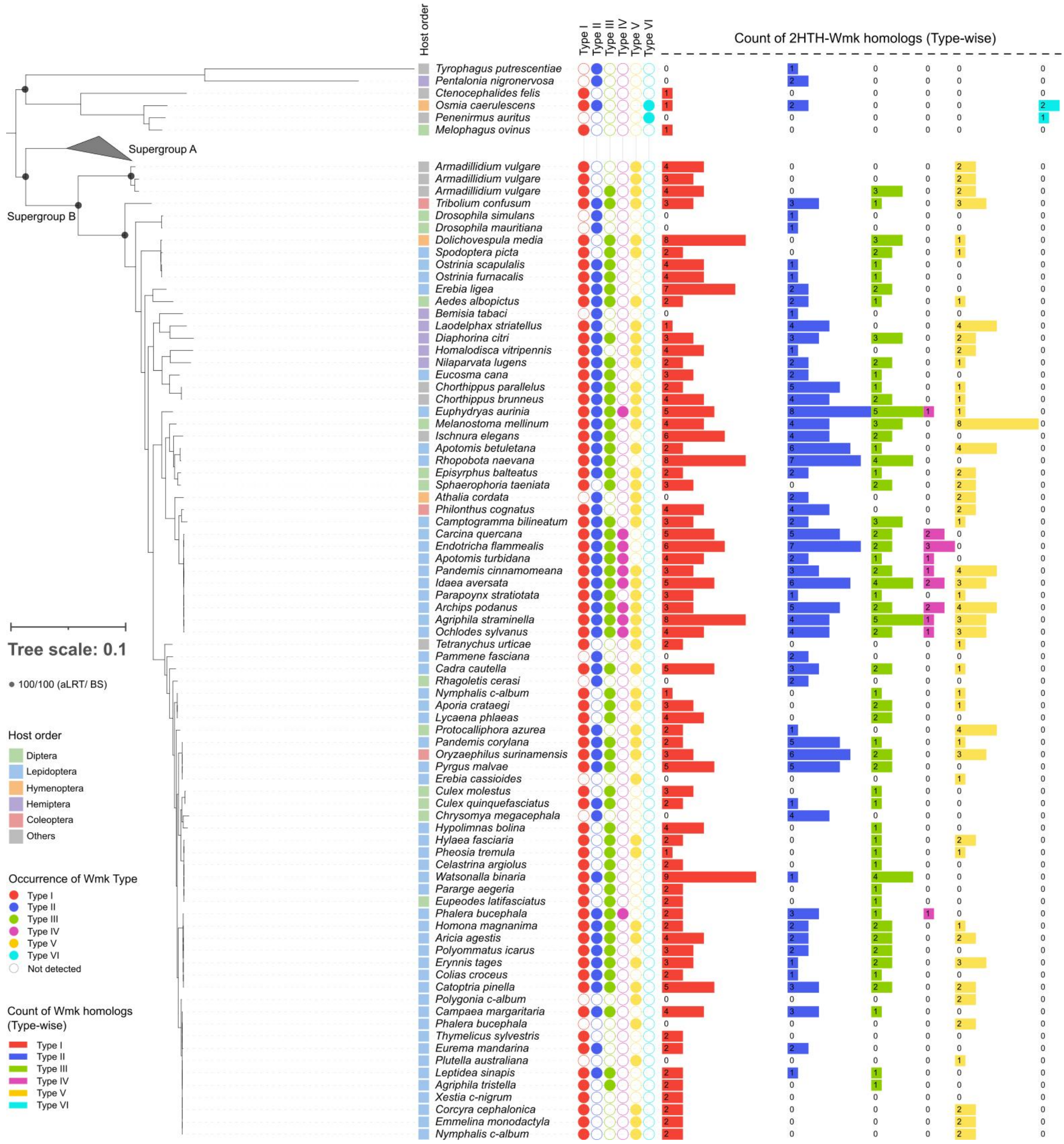

Figure S6. Pruned global ML phylogeny of *Wolbachia* retaining the 203 genomes that harbor at least one full-length Wmk homolog (Type I–VI). The original global phylogeny was reconstructed from 251 genomes. The tree here predominantly represents Supergroup B strains, along with six members of Supergroups E and F (placed towards the top of the map). The clade corresponding to Supergroup A is collapsed here; an expanded view is provided in Fig. 4A. Node support values are shown as approximate likelihood ratio test and ultrafast bootstrap percentages (aLRT/BS) based on 1000 replicates, with support values displayed for deeper nodes only. Tip labels indicate host taxonomic order , host species name, and the presence and copy number of full-length Wmk homologs spanning Type I–VI. Unfilled circles represent absence of Wmk homolog of corresponding type.
